# Assessing the severity of AstraZeneca COVID-19 vaccine-related side effects through pulse spectrum analysis

**DOI:** 10.1097/MD.0000000000037132

**Published:** 2024-02-09

**Authors:** Chen-Kai Liao, Shun-Ku Lin, Hsin Hsiu

**Affiliations:** a Graduate Institute of Applied Science and Technology, National Taiwan University of Science and Technology, Taipei, Taiwan; b Department of Chinese Medicine, Taipei City Hospital, Renai Branch, Taipei, Taiwan; c Institute of Public Health, National Yang Ming Chiao Tung University, Taipei, Taiwan; d Graduate Institute of Biomedical Engineering, National Taiwan University of Science and Technology, Taipei, Taiwan.

**Keywords:** AstraZeneca, blood pressure waveform, COVID-19, Fourier transform, side effect

## Abstract

AstraZeneca (AZ) vaccine is one of the most common vaccines against COVID-19 used globally. However, adverse reactions post-vaccination have been reported, including severe symptoms and cases of sudden death within several hours. Therefore, this study aimed to establish a database of spectral characteristics of blood pressure waveforms (BPWs) for the AZ vaccine and analyze reactions after vaccine administration using objective physiological signal and symptom analyses for identifying potential differences between heavy and slight groups defined in the study. In total, 24 participants were enrolled in the case-control study. BPW measurements were acquired pre- and post-vaccination. A questionnaire survey on side effects was conducted 5 days after vaccination. The related spectral characteristics of heavy and slight groups were acquired after Fourier transform analysis. Four types of harmonic indexes from BPW signals, including amplitude proportion (C_*n*_), coefficient of variation of C_*n*_ (CV_*n*_), phase angle (P_*n*_), and standard deviation of P_*n*_ (P_*n*__SD), were derived. The characteristics of harmonic indexes of arterial BPW for the AZ vaccine were in C_6_ (*P* = .011), CV_2_ (*P* = .027), P_5_ (*P* = .009), and P_2__SD (*P* = .027) on the radial pulse. C_5_ (*P* = .037), C_8_ (*P* = .007), C_9_ (*P* = .037), CV_5_ (*P* = .015), CV_8_ (*P* = .005), and CV_9_ (*P* = .028) were significantly different at posttest between heavy and slight groups. In both pretest or posttest, C_8_ was almost significantly different between slight and heavy groups. More parameters changed significantly post-vaccination, with more severe side effects. Most average values of posttest/pretest of CV_*n*_ and P_*n*__SD in the slight group exceeded 100%. All average values of posttest/pretest of CV_*n*_ and P_*n*__SD in the heavy group were smaller than 100%. This approach may enable prediction of the risk of reactions post-vaccination to determine suitability of the AZ vaccine and evaluation of side effect severity in vaccinated individuals using pulse analysis to ensure relevant precautions are taken.

## 1. Introduction

The vaccine AZD1222 (AstraZeneca [AZ]) was the first vaccine distributed by the COVID-19 Vaccines Global Access (COVAX)^[1]^ in response to the spread of the coronavirus disease 2019 (COVID-19). In a Phase III clinical study of AZ comprising 24,422 participants, the average efficacy rate reached 76.0% after the first dose and 81.3% following the second dose.^[2]^ Although the efficacy rate was less than that reported in Phase III clinical studies of the Pfizer-BioNTech COVID-19 and Moderna COVID-19 vaccines published in the same period, the AZ vaccine can be stored in a refrigerator at 2 to 8°C for 6 months, which has the advantages of easy storage and transportation. Moreover, it is considerably cheaper than other COVID-19 vaccines and thus considered a widely accessible and convenient vaccine to promote mass vaccinations globally.

Notably, side effects may occur after administration of vaccines, including the AZ vaccine. The common side effects include pain, redness and swelling at the injection site, fatigue, headache, myalgia, feverishness, fever, chills, arthralgia, and nausea. Even some rare cases of delayed cutaneous adverse reactions that had not been described in the AZ clinical trials were reported.^[3,4]^ Suspected severe symptoms include thrombosis with thrombocytopenia syndrome, cerebral venous sinus thrombosis without thrombocytopenia, myocarditis, pericarditis, Guillain–Barré syndrome, idiopathic thrombocytopenic purpura, and anaphylaxis.^[5]^ Several countries in Europe have suspended the use of the AZ vaccine owing to rare but serious conditions such as thrombosis with thrombocytopenia syndrome, and sudden death within hours or days after vaccination.^[6]^ Although it remains unclear from current clinical evidence whether these effects are directly related to vaccination, these outcomes have caused suspicion and deterrence among the public.

Pulse diagnosis in traditional Oriental medicine is a major feature of diagnostics, which is distinct to various diagnostic methods in Western medicine. However, quantification of pulse diagnosis remains challenging, and transferring the skills of predecessors is a long process, despite direct teaching and learning between the teacher and apprentice. It is even more difficult to learn pulse diagnosis solely from descriptions in books. In modern times, data analysis after harvesting the radial artery of the patient hand may solve this dilemma. In this regard, responses after vaccine administration may be predicted or evaluated using noninvasive pulse wave analysis as a research tool.

Vascular changes affect the transmission of the arterial pulse and its contours. Blood pressure waveform (BPW) indexes may provide information on changes in vascular properties and blood flow.^[7]^ Therefore, pulse wave analysis may be helpful for the diagnosis of cardiovascular-related diseases. In addition to time-domain BPW analysis, frequency-domain BPW analysis is another method to describe the pulse profile.^[8]^ Harmonic analysis is particularly suitable for measuring BPW signals because pulse beats are quasi-periodic. This method has been applied for many diseases, such as hypertension,^[9]^ stroke,^[10,11]^ liver cirrhosis,^[12]^ polycystic ovary syndrome,^[7]^ breast cancer,^[13]^ and type 2 diabetes.^[14]^ Clinical research suggests that herbal medicine affects specific harmonic spectra.^[15–19]^ Moreover, these spectra have been assessed in Western medicine.^[20,21]^

Compared with healthy controls, young patients infected with SARS-CoV-2 have significantly reduced vascular function and increased arterial stiffness.^[22]^ Arterial stiffness is a detrimental risk factor for COVID-19, and its all-cause mortality has an independent prognostic value.^[23]^ Various risk factors for COVID-19 are also associated with atherosclerosis, such as age,^[24]^ type 2 diabetes,^[25–27]^ hypertension,^[28]^ and chronic kidney disease.^[29–32]^ Additionally, among the common side effects of vaccination, fever alters the transmission of pulse waves, and pain may change vascular stiffness. For instance, increased systolic and pulse pressures in headache are related to arterial stiffness, and the degree of headache may be reduced by modulation of the baroreflex arch.^[33]^ COVID-19, its risk factors, and common side effects after vaccination will affect BPW; hence, the response after vaccine administration can be predicted or evaluated using convenient and noninvasive tools. The objectives of this study were 2-fold. First, the study aimed to establish a database of spectral characteristics of pulse waves for the AZ vaccine. Second, the study aimed to predict whether participants experienced heavy or slight discomfort by analyzing the human body reaction after vaccine administration using objective physiological signal and questionnaire analysis. The study examined whether and which of these parameters were significantly different between slight and heavy groups. This approach may help in predicting the risk of reactions post-vaccination to determine the suitability of the AZ vaccine and evaluating the severity of side effects in vaccinated individuals using pulse analysis to ensure relevant precautions are taken.

## 2. Materials and methods

### 2.1. Participants

In total, 24 participants between 20 and 85 years of age who received the AZ vaccine in the left upper arm were recruited from the Bioelectronic Application and Research Laboratory, National Taiwan University of Science and Technology between January 2022 and June 2022. Individuals who received other brands of COVID-19 vaccine were excluded. To avoid bias, individuals who had a stroke history, were pregnant, could not maintain a fixed posture for 1 minute when sitting, and were currently experiencing a cold were also excluded.

The study received approval by the Research Ethics Committee, National Taiwan University (REC permit No. 202112EM002). All participants received the recruitment news from the advertisement of Bio Electronic Application and Research Laboratory, National Taiwan University of Science and Technology, and signed consent forms before initiation of the study. Most participants lived in northern Taiwan and were recruited voluntarily.

### 2.2. Procedures

**Figure 1.**
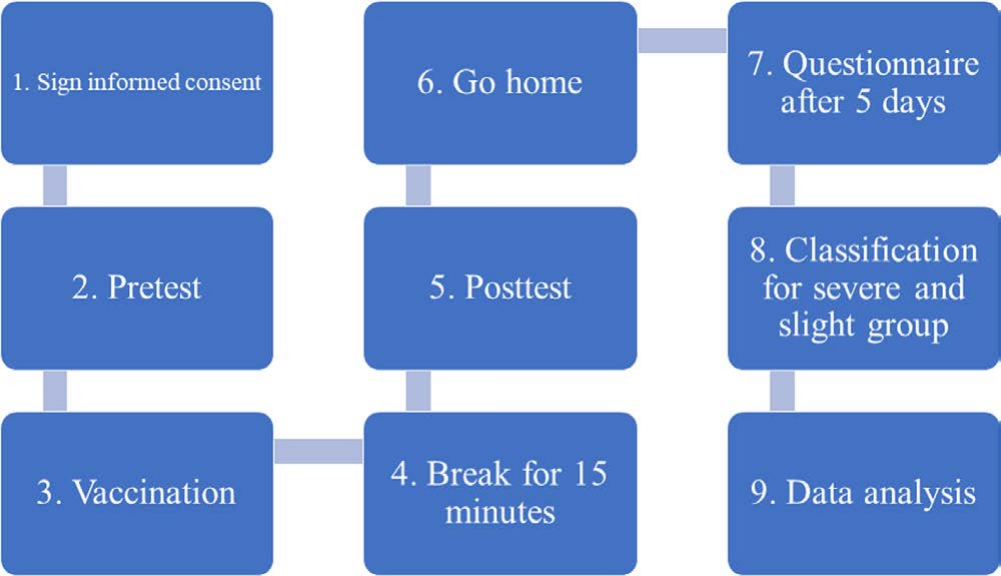
Study flowchart.

All enrolled participants met the study selection criteria. Participants were recruited during the daytime (8 am to 6 pm). Measurements were conducted at public sites maintained at 24 to 27°C. A pressure transducer (KFG-2-120-D1-11, Kyowa) sampled at 1024 Hz was used to collect radial pulse signals from participants.^[34,35]^ At 5 to 30 minutes before the injection, participants were measured on the right radial artery in a seated position while staying relaxed and maintaining natural breathing during the experiment to avoid motion artifacts. A second pulse wave measurement was obtained after a 15-minute rest according to regulations. Five days after the vaccination, a side effect questionnaire was conducted to identify the actual reaction after the vaccination. The contents of the questionnaire included pain and swelling at the injection site, fatigue, feverishness, fever (>38°C), chills, headache, nausea, arthralgia, and myalgia. Fast Fourier transform was performed on pretest and posttest measurement data on the day of vaccine administration. The spectral characteristics of the AZ vaccine were inferred by analyzing whether harmonics differed significantly between pretest and posttest. Participants were divided into heavy and slight groups according to the side effect questionnaire. Participants with a fever or those who indicated “yes” to 5 or more of the 9 items on the questionnaire were allocated to the heavy group. Other participants were assigned to the slight group. The related spectral characteristics of heavy and slight groups were acquired after Fast Fourier transform analysis, which enables evaluation of the severity of side effects before and after vaccination. The study flowchart is presented in Figure 1.

### 2.3. Harmonic analysis

A pressure transducer was used to measure BPW from the radial artery pulse at a 1024 Hz sampling rate for 1 minute, and 61,440 raw data points were acquired per measure. Four types of harmonic indexes from the BPW signal, including amplitude proportion (C_*n*_), coefficient of variation of C_*n*_ (CV_*n*_), phase angle (P_*n*_), and standard deviation of P_*n*_ (P_*n*__SD), were derived.

Each pulse wave defined between footpoints was represented using the following finite series^[34–37]^:


$$\begin{array}{l} x\left(t\right)=\frac{A0}{2}+\left\{\underset{n=1}{\overset{\frac{k}{2}}{\sum }}\;A_{n}cosn\omega t_{s}+\underset{n=1}{\overset{\frac{k}{2}}{\sum }}\;B_{n}sinn\omega t_{s}\right\} \end{array}$$

*A*_*n*_ and *B*_*n*_ are the Fourier coefficients of the pulse, which were calculated as follows:


$$\begin{array}{l} An=\frac{2}{k}\underset{s=0}{\overset{k}{\sum }}\;x_{s}cosn\omega t_{s}\;\left(\;for\;n=0,1,\ldots ,\frac{k}{2}\right) \end{array}$$

$$\begin{array}{l} Bn=\frac{2}{k}\underset{s=0}{\overset{k}{\sum }}\;x_{s}sinn\omega t_{s}\;\left(\;for\;n=0,1,\ldots ,\frac{k}{2}\right) \end{array}$$

where *ω* and *t*_*s*_ represent angular frequency and sampling time interval, respectively. The amplitude (Amp_*n*_) and phase angle (P_*n*_) of each harmonic of the pulse harmonic spectrum were then calculated as $Amp_{n}=\sqrt{A_{n}^{2}+B_{n}^{2}}$ and P_*n*_ = arctan (Bn/An). The amplitude proportions (C_*n*_ values) for each pulse were calculated as Amp_*n*_/Amp_0_ × 100%, for n = 1 to 10. CV_*n*_ was then calculated as the coefficient of variation of C_*n*_, and P_*n*__SD was calculated as the SD of P_*n*_. MATLAB (MathWorks, Natick, MA, US) was used to conduct the signal process. SPSS (SPSS Inc., Chicago, IL, US) was used to perform all statistical analyses. The Mann–Whitney U test and Wilcoxon signed-rank test were performed to analyze whether differences were significant. *P* < .05 was considered statistically significant.

### 2.4. Information processing

For information processing, the features of pulse signals were collected from the results of the signal-processing stage described above to yield 40 indexes for each pulse: C_*n*_, CV_*n*_, P_*n*_, and P_*n*__SD values for n = 1 to 10.

## 3. Results

Table 1 presents the clinical and demographic characteristics of participants. No significant differences were observed in sex and age between the slight and heavy groups. In total, 73% of the first-dose participants and 23% of the second-dose subjects were allocated to the heavy group. Side effects reported after the second dose were milder and less frequent than the first dose in the study.

**Table 1:** Clinical and demographic characteristics of slight and heavy groups.

Variables	Slight	Heavy	*P* value
N	13 (3 first dose and 10 second dose)	11 (8 first dose and 3 second dose)	
Age (yr)	39.91 ± 12.73	47.72 ± 14.01	.167
Sex			.562*
Male	5 (38.46%)	3 (27.27%)	
Female	8 (61.54%)	8 (72.73%)	

Data are expressed as mean ± standard deviation values. All *P* values were calculated by 2-tailed Student *t* test except asterisk symbol by chi-square tests.

**Figure 2.**
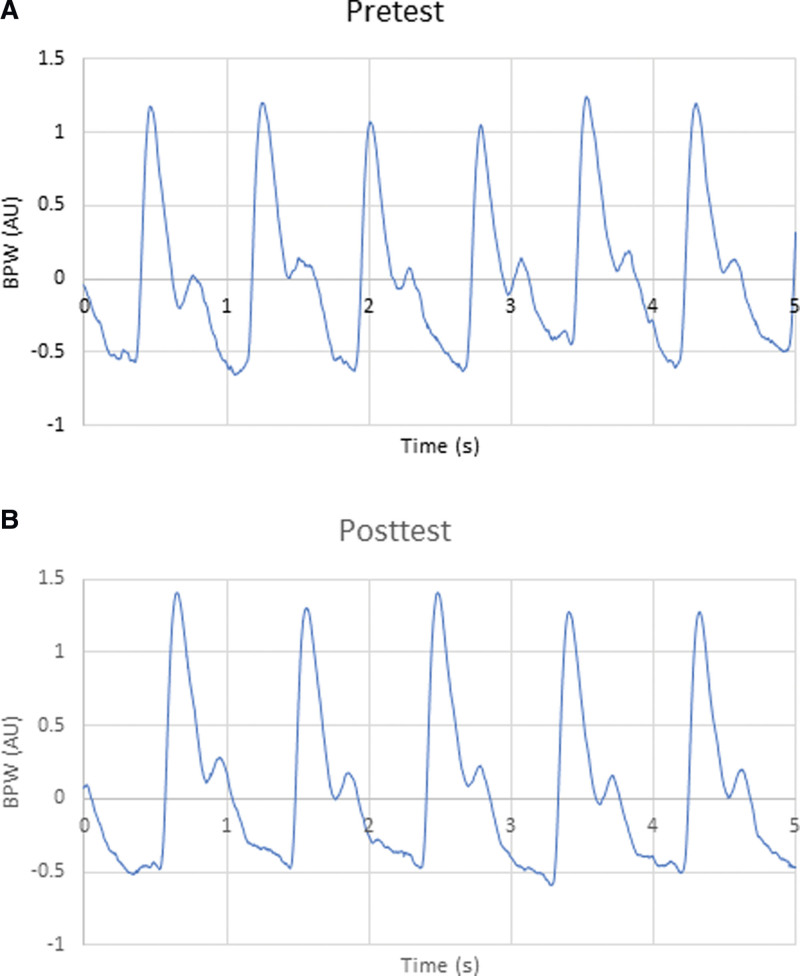
Pretest and posttest BPWs in the time domain. BPWs = blood pressure waveforms.

**Figure 3.**
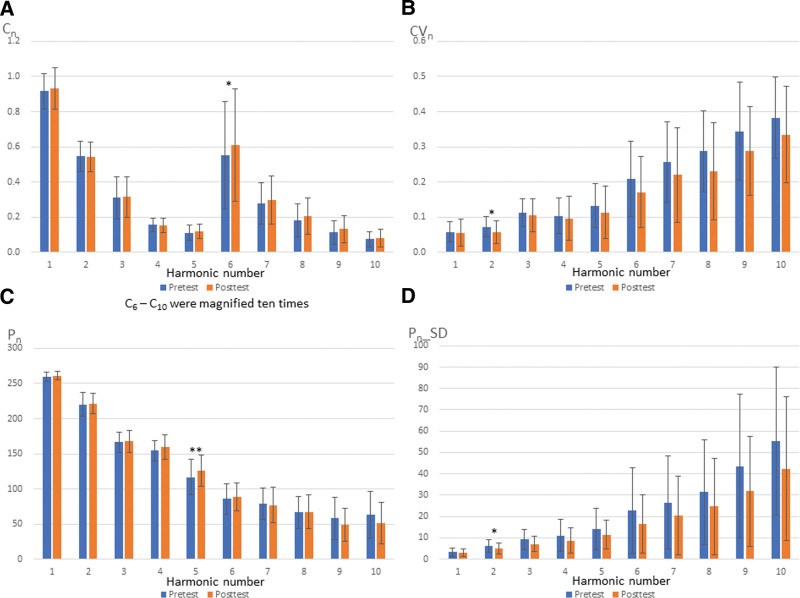
Pretest and posttest BPWs. BPWs = blood pressure waveforms.

**Figure 4.**
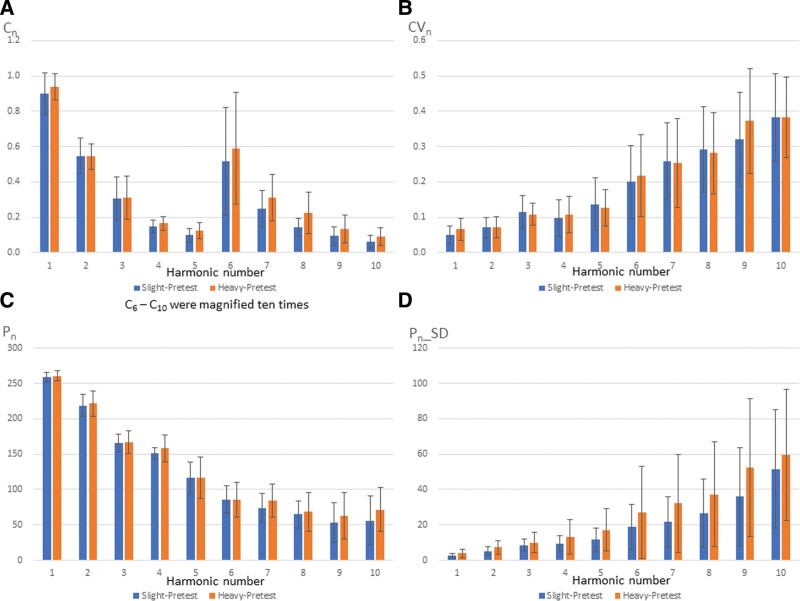
Comparison of pretest in slight and heavy groups.

Pretest and posttest BPWs in the time domain are presented in Figure 2. The spectrum analysis is transformed from the time domain by Fourier transform so that the results can be explained precisely. Significant differences were observed between pretest and posttest BPWs in all participants. The *P* values for C_6_ (*P* = .011), CV_2_ (*P* = .027), P_5_ (*P* = .009), and P_2__SD (*P* = .027) indicated significant differences between BPWs (Fig. 3). These indexes represented the AZ vaccine spectrum characteristics. The comparison of pretest and posttest values in slight and heavy groups could predict the severity of side effects. Comparison of pretest values in slight and heavy groups revealed no significant differences in parameters (Fig. 4).

**Figure 5.**
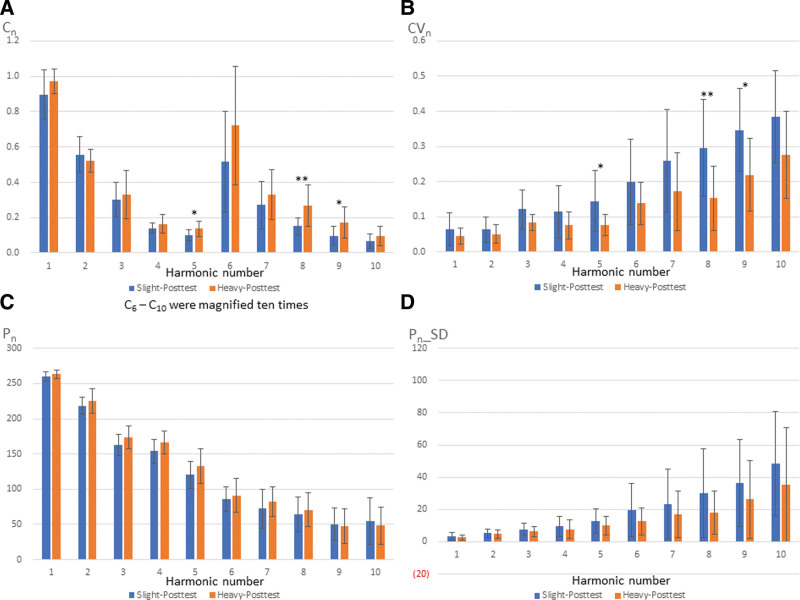
Comparison of posttest in slight and heavy groups.

**Figure 6.**
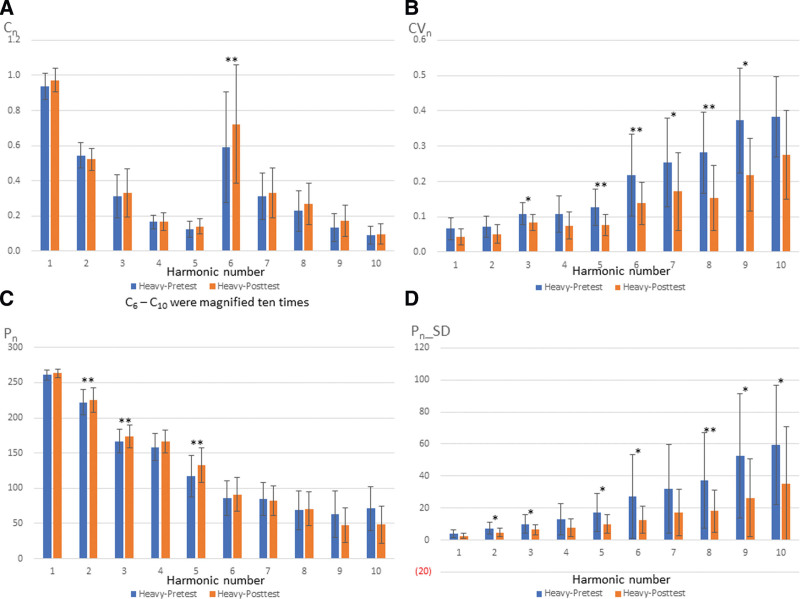
Comparison of pretest and posttest in heavy groups.

Figure 5 presents the comparison of posttest values in slight and heavy groups. Significant differences were observed in C_5_ (*P* = .037), C_8_ (*P* = .007), C_9_ (*P* = .037), CV_5_ (*P* = .015), CV_8_ (*P* = .005), and CV_9_ (*P* = .028). In the slight group, no significant difference was noted between pretest and posttest. However, 42.5% of the parameters in the heavy group were significantly different between pretest and posttest (Fig. 6). Significant differences were observed in C_6_ (*P* = .003), CV_3_ (*P* = .032), CV_5_ (*P* = .010), CV_6_ (*P* = .007), CV_7_ (*P* = .024), CV_8_ (*P* = .005), CV_9_ (*P* = .014), P_2_ (*P* = .010), P_3_ (*P* = .005), P_5_ (*P* = .003), P_2__SD (*P* = .014), P_3__SD (*P* = .019), P_5__SD (*P* = .042), P_6__SD (*P* = .032), P_8__SD (*P* = .005), P_9__SD (*P* = .024), and P_10__SD (*P* = .042). It revealed subjects in the heavy group differed in much more indexes of BPWs than those in the slight group.

## 4. Discussion

Through the objective pulse spectrum analysis, this study revealed significant differences in the characteristics of harmonic indexes of BPW in C_6_, CV_2_, P_5_, and P_2__SD after AZ vaccine administration. This indicated that the AZ vaccine resulted in a trend for a rise in C_6_ and P_5_, and a decrease in CV_2_, and P_2__SD. However, individuals may not have felt sick if their C_6_ and P_5_ increased or their CV_2_, and P_2__SD decreased.

In this study, comparison of posttest values in slight and heavy groups revealed significant differences in parameters such as C_5_, C_8_, C_9_, CV_5_, CV_8_, and CV_9_, suggesting the potential for more precise prediction compared to pretest. The findings suggest that these parameters may be key indexes to assess the risk of side effects and implement relevant precautions, underscoring the clinical value of these indexes in individuals who have received the AZ vaccine.

Although C_8_ (*P* = .056) did not meet the significance standard of 0.05 between slight and heavy groups at pretest, the *P* value of C8 was pretty close to a significant difference (Fig. 4). C_8_ was significantly different between slight and heavy groups at posttest, highlighting C_8_ as a key indicator for distinguishing the severity of side effects. C_8_ was higher in the heavy group than in the slight group, with a post-vaccination *P* value of .007, which was lower than the pretest value of 0.056, highlighting this parameter as a key index to assess the risk of side effects. Predicting whether a patient will have serious side effects before vaccine administration is essential. The severity of side effects can then be assessed to decide on whether the AZ vaccine should be administered. These findings suggest that subjects with higher C_8_ values will have a higher risk of side effects.

Comparison of pretest and posttest values in the slight group did not reveal significant differences in any of the parameters. However, 17 parameters were significantly different in the heavy group. This indicated that more parameters that were significantly altered in the heavy group after vaccination were associated with more severe side effects. Indeed, the more concurrent changes in the body, the more discomfort an individual would experience.

**Figure 7.**
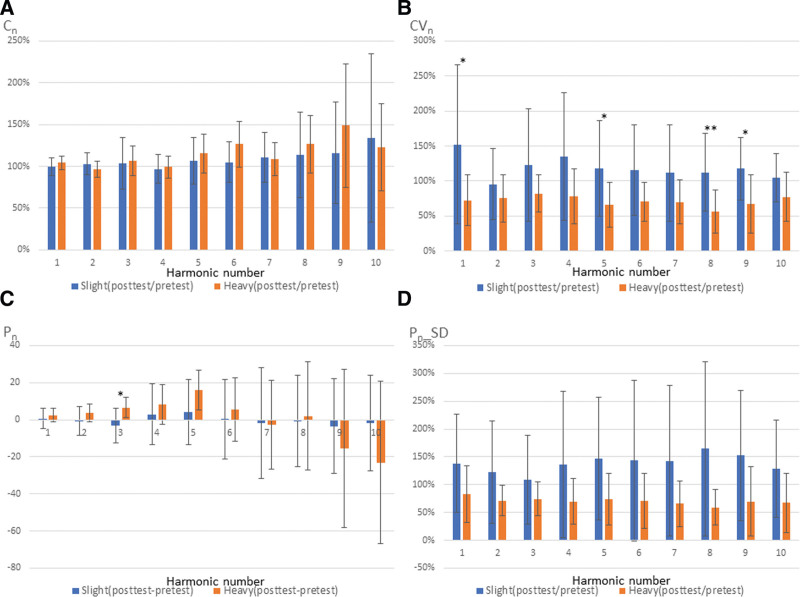
Posttest/pretest (or posttest—pretest) parameters in slight and heavy groups.

The difference between the 2 groups was larger at posttest than at pretest. With regard to the results when posttest values were divided by pretest values for C_*n*_, CV_*n*_, and P_*n*__SD, and posttest values were subtracted by pretest values in P_*n*_, it was expected a significant difference between the 2 groups. The statistical analysis of the study confirmed this speculation in Figure 7. The average values of posttest/pretest of CV_*n*_ and P_*n*__SD in the slight group were larger than 100%, with the exception of CV_2_. Moreover, the average values of posttest/pretest of CV_*n*_ and P_*n*__SD in the heavy group were smaller than 100%. This suggested that based on posttest/pretest of CV_*n*_ and P_*n*__SD, participants’ risk of side effects can be evaluated.

Table 2 presents a comparison of the proportion of side effects in a WHO report and those observed in the current study.^[38]^ Patients with fever were 3.7 times more common than in the WHO report. This may underscore the higher proportion of the heavy group in this study, since participants with fever were enrolled in the heavy group. However, adverse reactions reported after the second dose were milder and less frequent than those after the first dose (Table 2). This is consistent with the WHO report. The proportion of second dose in the WHO was 68.8% (8266/12021), which was higher than the proportion of 54.2% in this study. It is reasonable that most side effects in this study were more common than reported by the WHO, since the proportion of first dose was higher in this study.

**Table 2:** The comparison of the proportion of side effects in WHO report and the current study.

Proportion of side effect	WHO report (%)	The current study (%)	Slight group (%)	Heavy group (%)	First dose (%)	Second dose (%)
Injection site pain	54.2	91.7	84.6 (11/13)	100 (11/11)	100 (11/11)	84.6 (11/13)
Fatigue	53.1	66.7	53.8 (7/13)	81.8 (9/11)	63.6 (7/11)	69.2 (9/13)
Headache	52.6	54.2	23.1 (3/13)	90.9 (10/11)	90.9 (10/11)	23.1 (3/13)
Myalgia	44.0	45.8	23.1 (3/13)	72.7 (8/11)	63.6 (7/11)	30.8 (4/13)
Chills	31.9	25.0	7.7 (1/13)	45.5 (5/11)	54.5 (6/11)	0 (0/13)
Arthralgia	26.4	29.2	0 (0/13)	63.6 (7/11)	36.4 (4/11)	23.1 (3/13)
Feverishness	33.6	54.2	15.4 (2/13)	100 (11/11)	81.8 (9/11)	30.8 (4/13)
Fever (> 38°C)	7.9	29.2	0 (0/13)	63.6 (7/11)	54.5 (6/11)	7.7 (1/13)
Nausea	21.9	12.5	7.7 (1/13)	18.2 (2/11)	18.2 (2/11)	7.7 (1/13)

This study has several limitations. Most individuals have received different types of COVID-19 vaccines. Under the mixed vaccine policy in Taiwan, individuals can choose different brands of vaccines for each injection. Particularly, the AZ vaccine was not the public favorite choice in Taiwan. Individuals who received other brands of COVID-19 vaccine were excluded from this study, which resulted in the recruitment of fewer participants. Furthermore, some participants may have had chronic diseases such as diabetes mellitus, hypertension, and hyperlipidemia, whose effects were not assessed herein. Moreover, it was difficult to evaluate the coefficient of relevance and weight of these 9 side effects, which may have influenced the allocation of subgroups. Besides, individual parameters such as weight and height were not recorded completely might cause changes in pulse analysis, which was one of the limitations of this study.

## 5. Conclusions

This study demonstrated the characteristics of harmonic indexes of arterial BPW in the AZ vaccine. The research also identified a near difference in BPW analysis for C_8_ between heavy and slight groups at pretest. This approach may be applied to identify whether individuals should receive the AZ vaccine using BPW analysis. This may minimize potential side effects to avoid the risk of serious adverse reactions and promote public health. In particular, C_8_ is a key indicator to distinguish the severity of side effects between slight and heavy groups at pretest and posttest. Furthermore, more parameters were significantly altered after vaccination, with more severe side effects. Besides, all average values of posttest/pretest of CV_*n*_ and P_*n*__SD in the slight group were over 100% except CV_2_, and all average values of posttest/pretest of CV_*n*_ and P_*n*__SD in the heavy group were smaller than 100%. Those who have already received the vaccine can be evaluated for severity of side effects using pulse analysis to ensure necessary precautions are taken.

## Acknowledgments

The authors acknowledge the statistical assistance provided by biostatistics analyst, Tsung-Han Hsieh, Department of Research, Taipei Tzu Chi Hospital.

## Author contributions

**Conceptualization:** Hsin Hsiu.

**Data curation:** Chen-Kai Liao.

**Formal analysis:** Chen-Kai Liao.

**Investigation:** Chen-Kai Liao.

**Methodology:** Hsin Hsiu.

**Resources:** Shun-Ku Lin.

**Software:** Hsin Hsiu.

**Supervision:** Hsin Hsiu.

**Validation:** Hsin Hsiu.

**Writing – original draft:** Chen-Kai Liao.

**Writing – review & editing:** Shun-Ku Lin, Hsin Hsiu.
